# Toward High-Resolution Soil Moisture Monitoring by Combining Active-Passive Microwave and Optical Vegetation Remote Sensing Products with Land Surface Model

**DOI:** 10.3390/s19183924

**Published:** 2019-09-11

**Authors:** Kinya Toride, Yohei Sawada, Kentaro Aida, Toshio Koike

**Affiliations:** 1 Institute of Industrial Science, The University of Tokyo, Kashiwa, Chiba 277-8574, Japan; 2 Institute of Engineering Innovation, The University of Tokyo, Bunkyo-ku, Tokyo 113-8654, Japan; yohei.sawada@sogo.t.u-tokyo.ac.jp; 3 International Centre for Water Hazard and Risk Management (ICHARM), Tsukuba, Ibaraki 300-2621, Japan; aida-k677bt@pwri.go.jp (K.A.); koike@icharm.org (T.K.)

**Keywords:** active-passive, data assimilation, microwave remote sensing, soil moisture, downscaling, disaggregation, PALSAR, AMSR-E, MODIS

## Abstract

The assimilation of radiometer and synthetic aperture radar (SAR) data is a promising recent technique to downscale soil moisture products, yet it requires land surface parameters and meteorological forcing data at a high spatial resolution. In this study, we propose a new downscaling approach, named integrated passive and active downscaling (I-PAD), to achieve high spatial and temporal resolution soil moisture datasets over regions without detailed soil data. The Advanced Microwave Scanning Radiometer (AMSR-E) and Phased Array-type L-band SAR (PALSAR) data are combined through a dual-pass land data assimilation system to obtain soil moisture at 1 km resolution. In the first step, fine resolution model parameters are optimized based on fine resolution PALSAR soil moisture and moderate-resolution imaging spectroradiometer (MODIS) leaf area index data, and coarse resolution AMSR-E brightness temperature data. Then, the 25 km AMSR-E observations are assimilated into a land surface model at 1 km resolution with a simple but computationally low-cost algorithm that considers the spatial resolution difference. Precipitation data are used as the only inputs from ground measurements. The evaluations at the two lightly vegetated sites in Mongolia and the Little Washita basin show that the time series of soil moisture are improved at most of the observation by the assimilation scheme. The analyses reveal that I-PAD can capture overall spatial trends of soil moisture within the coarse resolution radiometer footprints, demonstrating the potential of the algorithm to be applied over data-sparse regions. The capability and limitation are discussed based on the simple optimization and assimilation schemes used in the algorithm.

## 1. Introduction

Soil moisture plays an important role in controlling the energy and water cycles in the land-atmosphere system [1,2,3]. However, soil moisture exhibits a high spatiotemporal variability, mainly dictated by soil texture, vegetation, meteorological forcing, and topography [4,5,6]. Due to the high variability, it is impractical to fully capture the soil moisture behavior in time and space by sparsely distributed ground-based point measurements. Therefore, there has been a compelling need to quantify soil moisture with high spatial and temporal resolutions for successful hydrological, meteorological, and agricultural applications [7].

Satellite remote sensing can measure soil moisture on a large spatial scale with consistent accuracy at a constant revisit interval. Among the remote sensing techniques which can measure soil moisture, passive microwave remote sensing systems have proven to be the most promising technique to measure soil moisture with shorter revisit times through sensing the dielectric properties of the soil [8,9]. However, major passive remotely sensed soil moisture products, such as the Advanced Microwave Scanning Radiometer 2 (AMSR2), the Soil Moisture Ocean Salinity system (SMOS), and the Soil Moisture Active and Passive system (SMAP), have coarse spatial resolutions (several tens of kilometers) for hydrological applications. As a consequence, many studies have explored downscaling or disaggregation methods for deriving fine resolution soil moisture from passive microwave observations through combining optical and thermal remote sensing observations [10,11]; soil surface attributes [12]; and higher frequency radiometer observations [13].

Active microwave sensors also detect surface soil moisture through the changes in the soil dielectric properties. For instance, the Advanced Scatterometer (ASCAT) can measure daily soil moisture with 25 km resolution [14]. Furthermore, synthetic aperture radars (SARs) achieve higher spatial resolutions but with long revisit intervals such as the phased array-type L-band SAR (PALSAR) and Sentinel-1 SAR. The combination of radar and radiometer observations is another approach to downscale soil moisture, which outperforms other approaches in accuracy and robustness [7]. To obtain the maximum benefit of using active and passive microwave sensors, SMAP was designed to carry L-band radar and radiometer instruments with resolutions of 3 km and 36 km, respectively [15]. Das et al. [16] proposed an active-passive retrieval algorithm, which estimates soil moisture with a 9 km resolution assuming the linear relationship between brightness temperature and backscattering. As the radar onboard SMAP stopped transmitting due to an anomaly in July 2015, it is no longer possible to combine SMAP active and passive observations. Nevertheless, Das et al. [17] utilized the active and passive data during the 2.5 months before the failure and demonstrated the potential of combining radar and radiometer data for monitoring high-resolution soil moisture.

As using radar and radiometer systems on the same satellite is not currently possible, the combined use of different satellite datasets has significantly become important. Although several studies have utilized both active and passive data to obtain higher resolution products [18,19,20,21], they have focused on snapshot downscaling but not continuous time series. Therefore, these methods require active and passive sensors to concurrently overpass the target region at every time step, which is impractical for the long revisit interval of SARs.

Recently, data assimilation-based downscaling techniques have been developed to downscale coarse passive microwave data by using a high-resolution land surface model. The advantages of the model-based approach are that it can be applied for datasets with any revisit intervals and data loss due to cloud coverage, and it is capable of assimilating various types of observations. Draper et al. [22] assimilated 25 km ASCAT soil moisture and 25 km resampled AMSR for Earth Observing System (AMSR-E) soil moisture into a 25 km land surface model. Similarly, Kolassa et al. [23] assimilated ASCAT and AMSR-E soil moisture datasets into a land surface model and validated against point-scale measurements over various sites in the United States. These studies, however, focused on the joint assimilation of radar and radiometer datasets and did not downscale radiometer data. Lievens et al. [24] assimilated 36 km SMAP brightness temperature and aggregated 9 km Sentinel-1 SAR backscatter [25] observations into a 9 km land surface model. They used the three-dimensional ensemble Kalman filter that considers the scale difference between the SMAP observations and the land surface model. Some studies also used similar data assimilation techniques to downscale passive microwave observation without radar data [26,27,28]. However, the main drawback of these studies is the requirements of meteorological and land surface parameters at a high spatial resolution to simulate land surface models, restricting the applications to data-sparse regions.

This study investigates a new approach to estimate high spatial and temporal resolution soil moisture data by fusing AMSR-E and PALSAR data into a land surface model. The objective of this study is to develop a new downscaling algorithm, named integrated passive and active downscaling (I-PAD), that is applicable to regions without dense land surface datasets. The PALSAR and moderate-resolution imaging spectroradiometer (MODIS) data are utilized as high-resolution initial conditions and constraint to estimate land surface parameters at a high resolution (1 km). Then, 25 km AMSR-E C-band (6.9 GHz) and K-band (18.7 GHz) observations are assimilated with a simple algorithm that accommodates the spatial resolution difference between the model and observations. The proposed I-PAD method is evaluated at lightly vegetated regions using ground observations, PALSAR observations, and a well-validated hydrological model.

**Figure 1:**
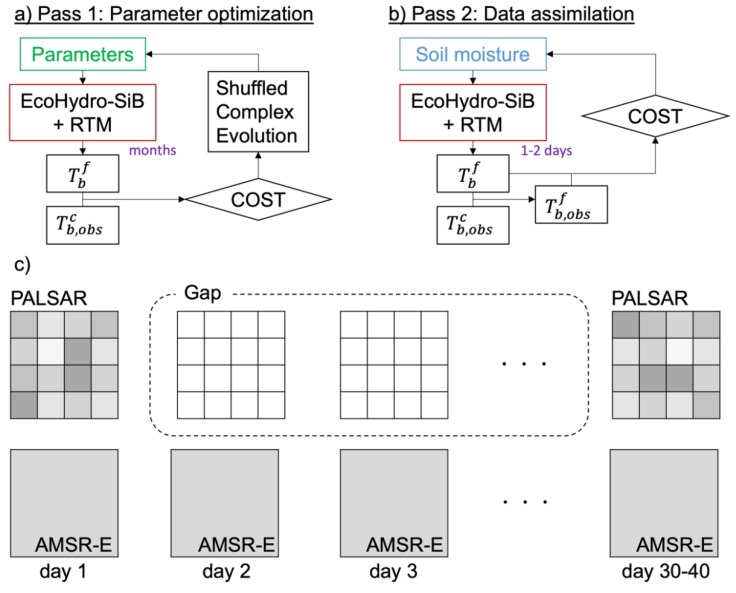
Flow chart of the integrated passive and active downscaling (I-PAD) system. (**a**) Parameter optimization process and (**b**) data assimilation process. The cost functions are calculated based on coarse resolution observed brightness temperature $T_{b,obs}^{c}$ and fine resolution simulated brightness temperature $T_{b}^{f}$. The superscripts *c* and *f* represent coarse and fine resolutions, respectively. (**c**) The target of the current study.

The developed algorithm is based on the Coupled Land and Vegetation Data Assimilation System (CLVDAS) [29]. This is a dual-pass land data assimilation system that consists of parameter optimization and data assimilation processes. The data assimilation step uses a simple algorithm to assimilate coarse AMSR-E brightness temperature observations into a high-resolution land surface model assuming a linear decomposition of radiometer data (Figure 1). The benefit of using the simple algorithm is that it does not require as high computational resources as ensemble methods, which is crucial for global applications. This algorithm is designed to simulate from an initial PALSAR observation to the subsequent PALSAR observation to fill the gap between PALSAR and AMSR-E observations as illustrated in Figure 1c.

## 2. Study Area and Datasets

### 2.1. Study Areas and In-Situ Observations

**Figure 2:**
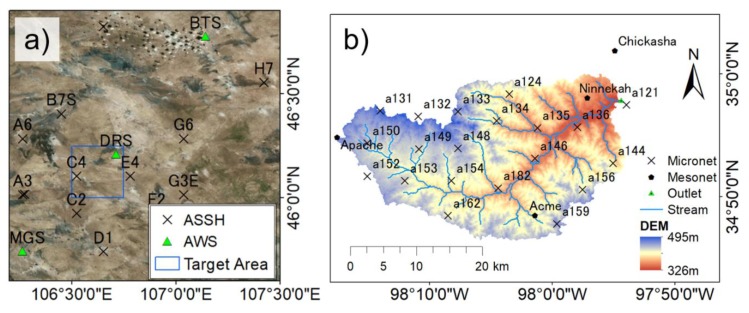
Maps of the target areas and observation stations. (**a**) CEOP/Mongolia reference site; (**b**) Little Washita Basin. Digital elevation model (DEM) was obtained from the National Elevation Dataset provided by the United States Geological Survey.

We selected two observation sites to evaluate the performance of I-PAD. The first experiment was conducted at the flat semiarid CEOP Mongolia reference site (Figure 2a), comprising 13 automatic stations for soil hydrology (ASSH) and three automatic weather stations (AWS) [30]. Soil moisture and soil temperature were measured at depths of 3 cm and 10 cm at ASSH. Wind, temperature, humidity, pressure, and precipitation were measured at AWS, in addition to soil moisture and soil temperature. In this study, the target area was set from 106.50°E to 106.75°E longitude and from 46.00°N to 46.25°N latitude, which contains the AWS DRS site and the ASSH C4 site (blue box in Figure 2a). Soil moisture measurements at 3 cm depth were used to validate simulated surface soil moisture. The measurements were taken by time domain reflectometry (TDR) probes that were set horizontally at 3 cm depth and designed to measure the average volumetric water content from 1.5 to 4.5 cm depths [31]. Simulation periods were selected from June to September to avoid soil freezing and thawing processes, as the main interest of this study was in the spatial heterogeneity of soil moisture.

The second target area was selected at the Little Washita basin, located in Oklahoma, USA (Figure 2b). The Little Washita watershed is a well-known site for hydrological validations and applications, with a number of field campaigns undertaken, such as the Southern Great Plains Hydrology Experiments (SGP97 and SGP99) and the Soil Moisture Experiments (SMEX02, SMEX03, and SMEX04). The catchment encompasses an area of 603 km^2^ and is a sub-humid river basin with an average annual rainfall of 750 mm. This watershed was selected because of the existence of a moderate slope, which plays a key role for both wetting and drying processes after rainfall, and the abundant in-situ data for validation. Twenty agricultural research service (ARS) Micronet stations were available, which provided soil moisture at 5 cm, 25 cm, and 45 cm depths, rainfall, solar radiation, relative humidity, air temperature, and soil temperature data [32]. Four Oklahoma Mesonet stations (ACME, APAC, CHIC, and NINN) also provided rainfall, solar radiation, pressure, relative humidity, air temperature, soil temperature, and wind speed data [33,34]. In this study site, soil moisture measurements at 5 cm depth were used to validate simulated surface soil moisture. Hydra Probes (Stevens Water Monitoring Systems, Inc., Portland, OR, USA) are installed horizontally at 3–7 cm depths (average of 5 cm) and considered as the 0–5 cm average volumetric water content [35].

Only precipitation data were used as in-situ data to run I-PAD. At the Mongolia site, spatially homogeneous rainfall was used because only the AWS DRS site measured rainfall within the study area. In contrast, rainfall data at the ARS Micronet and Mesonet stations in Little Washita were interpolated to a resolution of 0.01° using the Inverse Distance Weighting (IDW) method [36].

### 2.2. Datasets

PALSAR is a multi-polarimetric L-band SAR with a spatial resolution of about 10 m, and a revisit interval of about 30–40 days. Soil moisture content was retrieved by PALSAR backscatters using the algorithm developed by Aida et al. [37] with a slope correction method. Although PALSAR has a polarimetry (HH/HV/VH/VV) mode, which is effective for separating the contributing factors in backscattering, its normal operation modes are fine-beam single polarization (FBS; HH or VV) and fine-beam dual polarization (FBD; HH/HV or VV/VH) modes. This method utilizes the information of a limited number of the polarimetry mode to retrieve surface soil moisture from the other frequent observation modes by assuming the homogeneity in soil characteristics within the image swath and the stationarity in parameters during the observation period (see Appendix A for further details). Table 1 shows the dates of PALSAR soil moisture retrieved in this study at each target area. The retrieved data were spatially averaged to a resolution of 0.01° (about 1 km) to use for model initial conditions. Since the PALSAR L-band sensor has deeper penetration depth than the model surface layer (5 cm), the mean of the estimated soil moisture was then bias-corrected by linear scaling.

For passive microwave remote sensing, brightness temperature observations of AMSR-E on the EOS Aqua satellite were employed. AMSR-E is a multi-channel instrument with frequencies of 6.9, 10.7, 18.7, 23.8, 36.5, and 89 GHz in horizontal and vertical polarization. In this study, 6.9 GHz (C-band), which is sensitive to soil moisture, and 18.7 GHz (K-band), which is sensitive to vegetation water content were used following the study by Sawada and Koike [38]. The penetration depth of these two frequencies is typically 1-2 cm with deeper penetration for C-band. The spatial resolutions are 75 × 43 km at 6.9 GHz and 27 × 16 km at 18.7 GHz. The brightness temperature from the descending orbit (1:30 am) at almost daily resolution was resampled at a resolution of 0.25° (about 25 km). In addition, we obtained the AMSR-E Level 3 daily soil moisture product [39] for the validation of the I-PAD results. This product can be obtained through the Globe Portal System website (https://gportal.jaxa.jp/gpr/?lang=en). Please note that the AMSR-E soil moisture product was only used for validation purpose and not used for assimilation.

Vegetation is one of the key drivers of soil moisture. Leaf area index (LAI), which is one of the state variables in the land surface model employed in this study, were obtained from MODIS (MCD15A2) to use high spatial resolution vegetation information for initial conditions. It is the 8-day product with 1 km resolution, which collects the best observation to remove clouds effects. The obtained data were resampled at a resolution of 0.01° with the nearest-neighbor method.

Meteorological forcing data (rainfall, solar radiation, longwave radiation, air temperature, specific humidity, air pressure, and wind speed) are necessary to run I-PAD. Although in-situ data were used with respect to rainfall to reduce error sources, the final goal of this study was to apply I-PAD globally without relying on in-situ observations. Therefore, the Global Land Data Assimilation System (GLDAS) [40] data were used for other forcing data while some finer resolution datasets are available for the Little Washita basin such as the North American Land Data Assimilation System Phase 2 (NLDAS-2) [41,42]. Since GLDAS data are available at 0.25° resolution, we assumed uniform forcing over the modeling domain. In addition, surface soil moisture of GLDAS was used to bias-correct PALSAR soil moisture, and GLDAS land surface data were used as initial conditions except for surface soil moisture and LAI.

## 3. Methods

I-PAD is a dual-pass downscaling algorithm (Figure 1) based on the Coupled Land and Vegetation Data Assimilation System (CLVDAS) [29,38,43], which is an upgraded version of the Land Data Assimilation System of the University of Tokyo (LDAS-UT) [44]. LDAS-UT was the first land data assimilation system that utilized a dual-pass assimilation technique to assimilate AMSR/AMSR-E low-frequency observations. CLVDAS was then further developed by introducing vegetation dynamics and simultaneous assimilation of soil moisture and LAI. The grid size of LDAS-UT and CLVDAS depends on the observed brightness temperature scale, whereas I-PAD can have a grid size smaller than the scale of the observed brightness temperature. The simultaneous assimilation of soil moisture and LAI was not adopted in this study.

The I-PAD model used high spatial resolution soil moisture (aggregated to 0.01° resolution) from PALSAR and LAI from MODIS as initial conditions. In the first step, model parameters at 0.01° resolution were optimized using the AMSR-E brightness temperature with a long-time window (several months) as shown in Figure 1a. Then, in the assimilation step, surface soil moisture in the fine model grids (0.01° resolution) was adjusted with the coarse AMSR-E brightness temperature (0.25° resolution) on a daily basis considering the scale discrepancy (Figure 1b). In this study, 6.9 GHz and 18.7 GHz AMSR-E observations were used in both steps without considering the difference in penetration depth. This downscaling approach incorporated EcoHydro-SiB [45] as a land surface model to forecast land surface variables, and a radiative transfer model (RTM) [46] to calculate brightness temperature. A core system operated the two applications seamlessly. EcoHydro-SiB, the RTM, and the structure of the dual-pass system are explained in the following sections.

### 3.1. Land Surface Model

EcoHydro-SiB [45] was employed as a land surface model to forecast land surface variables in this study. It is an improved version of Hydro-SiB [47] with a dynamic vegetation scheme that explicitly simulates vegetation growth and senescence. The model has a multiple sublayer structure to calculate vertical interlayer flows based on the one-dimensional Richards equation. The first layer depth of the model was set to 5 cm in this study. The EcoHydro-SiB was applied in various climatological regions and showed reliable accuracy in calculating water and vegetation dynamics, and drought indices [43,45,48]. The model parameters include hydrological parameters such as saturated hydraulic conductivity, porosity, and van Genuchten’s water retention curve parameters [49], ecological parameters such as turnover rate of leaves, water-related stress parameters, and temperature-related stress parameters.

### 3.2. Radiative Transfer Model

The RTM [46] calculates microwave brightness temperature based on the surface soil moisture, surface temperature, canopy temperature, and vegetation water content estimated by EcoHydro-SiB. In this model, the brightness temperature at 6.9 and 18.7 GHz with horizontal and vertical polarization was calculated based on the τ-ω model [50] with the surface reflectivity estimated by the physically-based advanced integral equation model (AIEM) incorporating a shadowing effect. The effects of the atmosphere are minimal in these microwave regions and they were not considered in calculating AMSR-E brightness temperature. The parameters in the RTM are roughness parameters, soil texture parameters, and vegetation parameters.

### 3.3. Parameter Optimization Scheme

There are in total 19 parameters in EcoHydro-Sib and the RTM [38]. To make I-PAD applicable to data-sparse regions, these parameters are auto-calibrated by fitting estimated brightness temperature with the observed brightness temperature (Figure 1a). This step utilizes a long-time window (~months) by assuming that the parameters do not change dramatically over the period. The optimum parameter set at each grid point (*i, j*) is defined as that which minimizes the following cost function:

(1) $${\mathrm{COS}T}_{i,\;j}=\overset{T}{\underset{t=0}{\sum }}[{\left(T_{b,est}^{6.9V}{}_{i,\;j}-T_{b,obs}^{6.9V}\right)}^{2}+{\left(T_{b,est}^{6.9H}{}_{i,j}-T_{b,obs}^{6.9H}\right)}^{2}+{\left(T_{b,est}^{18.7V}{}_{i.j}-T_{b,obs}^{18.7V}\right)}^{2}+{\left(T_{b,est}^{18.7H}{}_{i,j}-T_{b,obs}^{18.7H}\right)}^{2}],\#$$

where $T_{b,est}^{6.9p}{}_{i,j}$ and $T_{b,est}^{18.7p}{}_{i,j}$ are the fine scale estimated brightness temperatures at the point (*i, j*) at frequencies of 6.9 GHz and 18.7 GHz, respectively, $T_{b,obs}^{6.9p}$ and $T_{b,obs}^{18.7p}$ are the coarse scale observed brightness temperatures, and p is the polarization (V, vertical; H, horizontal). T was set to 40–60 days in this study.

The shuffled complex evolution method [51] was used for searching the global optima for the parameters at each model grid point. The optimization was applied at each fine scale (0.01°) model grid point initiated from high-resolution PALSAR soil moisture and MODIS LAI using the coarse AMSR-E observations. Therefore, the difference between the model grid scale and the AMSR-E footprint was not considered in the optimization process. The optimized parameter sets were not directly validated in this study.

### 3.4. Assimilation Scheme

The assimilation algorithm was developed to connect high spatial and low temporal resolution SAR data, and low spatial and high temporal resolution radiometer data. The spatial resolution of CLVDAS depends on radiometer data and it does not consider heterogeneity inside model grids. To overcome this, we utilized PALSAR soil moisture and MODIS LAI data as initial conditions, which included high spatial resolution information, and maintained temporal fluctuation with AMSR-E data on a coarse scale.

**Figure 3:**
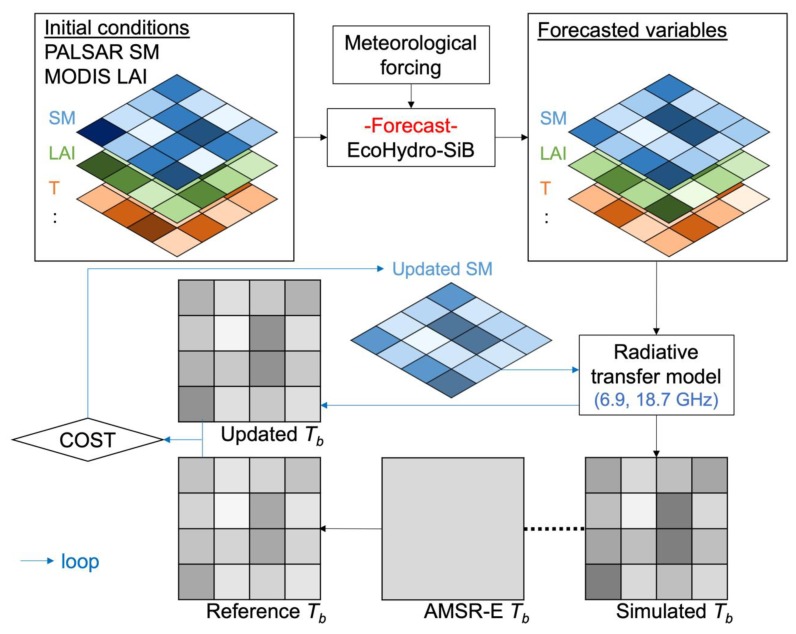
Schematic representation of the assimilation system. High-resolution initial conditions of soil moisture (SM) and leaf area index (LAI) are obtained from PALSAR and MODIS observations. Then, the land surface model EcoHydro-Sib estimates the evolution of land surface states until when AMSR-E has a brightness temperature (*T_b_*) observation. With the forecasted land surface variables, the radiative transfer model calculates brightness temperature at each grid point. The fine resolution reference brightness temperature is estimated by the coarse resolution observed brightness temperature and the fine resolution simulated brightness temperature by assuming linearity in microwave emission. In the assimilation loop, soil moisture at each grid point is updated to minimize the cost between the updated and reference brightness temperature.

The optimized parameters for each fine resolution grid point were used in this step to simulate high-resolution soil moisture dynamics. Figure 3 illustrates the structure of this assimilation system. When brightness temperature was observed by the passive sensor, soil moisture, vegetation, and other land surface variables estimated by EcoHydro-SiB were used as the RTM inputs for estimating the brightness temperature at 0.01° resolution. To compare with the observed brightness temperature at 0.25° resolution, we assumed the brightness temperature on a footprint scale can be approximated from the arithmetic mean of the brightness temperature on a fine scale [52,53]. Consequently, the reference brightness temperature at the fine scale was estimated by the following equations using the observed brightness temperature at the coarse scale:

(2) $$T_{b,ref}^{6.9V}{}_{i,\;j}=T_{b,est}^{6.9V}{}_{i,\;j}\frac{T_{b,obs}^{6.9V}}{\sum_{i,j}T_{b,est}^{6.9V}{}_{i,\;j}/N},\;\;T_{b,ref}^{18.7V}{}_{i,\;j}=T_{b,est}^{18.7V}{}_{i,\;j}\frac{T_{b,obs}^{18.7V}}{\sum_{i,j}T_{b,est}^{18.7V}{}_{i,\;j}/N},$$

where $T_{b,\;\;ref}^{6.9V}{}_{i,\;j}$ and $T_{b,\;\;ref}^{18.7V}{}_{i,\;j}$ are the reference brightness temperatures at the point (*i, j*) at frequencies of 6.9 GHz and 18.7 GHz, respectively. *N* is the total number of the grid points within the AMSR-E footprint. By using the reference brightness temperatures, the system searched for the optimum surface soil moisture at each grid point which minimizes the following cost function:

(3) $${\mathrm{COS}T}_{i,j}\;={\left(T_{b,est}^{6.9V}{}_{i,j}^{’}-T_{b,ref}^{6.9V}{}_{i,\;j}\right)}^{2}+{\left(T_{b,est}^{18,7V}{}_{i,j}^{’}-T_{b,ref}^{18.7V}{}_{i,\;j}\right)}^{2},$$

where $T_{b,est}^{6.9V}{}_{i,j}^{’}$ and $T_{b,est}^{18,7V}{}_{i,j}^{’}$ are the brightness temperatures at the point (*i, j*) estimated by the modified soil moisture at the frequency of 6.9 GHz and 18.7 GHz, respectively. This method assumed that there are no uncertainties in AMSR-E brightness temperature observations. The simple assimilation method was chosen to reduce the computational costs of EcoHydro-SiB and RTM that had to be solved at each grid point (25 × 25 points in this study). The assimilated soil moisture value was used as an input for the next time step calculation.

### 3.5. Experimental Design

I-PAD was driven from the days listed in Table 1 for about two months to simulate from an initial PALSAR observation to the subsequent PALSAR observation. Thus, this algorithm can be used to compensate for the low temporal resolution of PALSAR observations (Figure 1c). The surface soil moisture results were compared after the parameters were calibrated (hereafter optimized model or opt), and after soil moisture was assimilated (assimilated model or assim).

As the aim of this algorithm was to obtain a high spatial and temporal resolution soil moisture dataset by taking advantage of the active and passive sensors, it should be evaluated both spatially and temporally. For this purpose, we examined the algorithm using three sources. First, in-situ observations were used to evaluate the temporal variations in soil moisture. Second, the spatial distribution of soil moisture calculated by I-PAD was compared with the soil moisture maps obtained by PALSAR. The PALSAR images used for the evaluation were images taken subsequent to those used for the initial conditions (usually after 30–40 days). However, soil moisture retrieved from PALSAR observations has own uncertainties generated from the algorithm which may bias the evaluations. To mitigate this issue, WEB-DHM was additionally used to examine the spatial distribution of soil moisture. WEB-DHM is a hydrological model that couples Hydro-SiB and a geomorphology-based hydrological model (GBHM) to incorporate the topography effects on soil water redistribution processes [54]. We chose WEB-DHM as an additional independent reference because it calculates detail moisture movements using high-resolution topography, soil properties, land use, and vegetation data. We configured WEB-DHM and validated its soil moisture outputs over the Little Washita basin as shown in Appendix B. Please note that the third validation method was only applied in the Little Washita basin where the sufficient amount of data are available and the soil moisture estimation by WEB-DHM was intensively validated using two aircraft datasets obtained in SGP97 and SGP99 [54].

Time series of simulated surface soil moisture values were evaluated using skill metrics such as root-mean-square error (RMSE, m^3^ m^−3^), correlation coefficient (R), and mean bias (m^3^ m^−3^) against in-situ observations. With respect to spatial distributions, standard score normalization was done to compare with PALSAR observations which are sensitive to deeper soil depth.

## 4. Results

### 4.1. Mongolia

**Figure 4:**
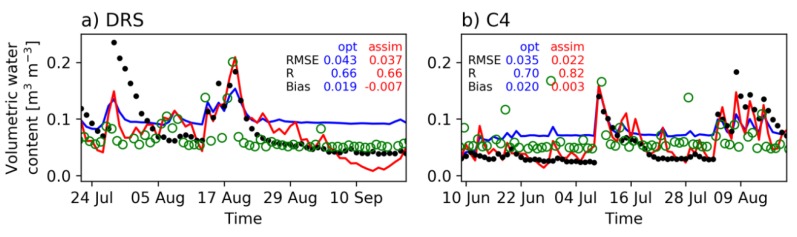
Time series of surface soil moisture (m^3^ m^-3^) from the optimized model (blue), assimilated model (red), observation (black dot), and AMSR-E soil moisture (green circle) at (**a**) DRS in 2009 and (**b**) C4 in 2010 in Mongolia. Skill metrics are indicated on each figure as the root-mean-square error (RMSE), correlation (R), and bias.

Figure 4 shows the comparison of the optimized model, assimilated model, and in-situ observation for daily surface soil moisture at DRS and C4 stations in Mongolia. The model results were obtained from the closest grid points to the stations. Although the optimized model overestimated soil moisture at both sites during the dry period, the RMSEs were about 0.04 m^2^ m^−2^ which satisfy the soil moisture accuracy requirement proposed in the SMAP mission [15]. The surface soil moisture estimation became even better after the assimilation of AMSR-E brightness temperature. The corresponding AMSR-E soil moisture products are also shown in green circles. Some discrepancies between the estimated and in-situ observed soil moisture were seen, such as the peak on 28 July 2009 at DRS. This may be due to the limitation of using uniform rainfall over the domain. However, the I-PAD estimate of the peak was better than the AMSR-E soil moisture, suggesting the positive effects of the parameter estimation initiated by PASLAR soil moisture. Conversely, the limitations of decomposing the single AMSR-E observation linearly to many grid points were detected. Because I-PAD fundamentally adjusts high spatial resolution soil moisture according to the mean brightness temperature over the domain, soil moisture at a single grid point could have extreme values as can be seen in the last days in Figure 4a. The results at C4 show that the assimilated model (I-PAD) estimated better soil moisture both for the lower limit and the peaks during early August when compared to the optimized model and AMSR-E soil moisture. Therefore, assimilating the AMSR-E brightness temperature had overall positive impacts on the simulation of soil moisture temporal variations as observed in the RMSEs and biases.

**Figure 5:**
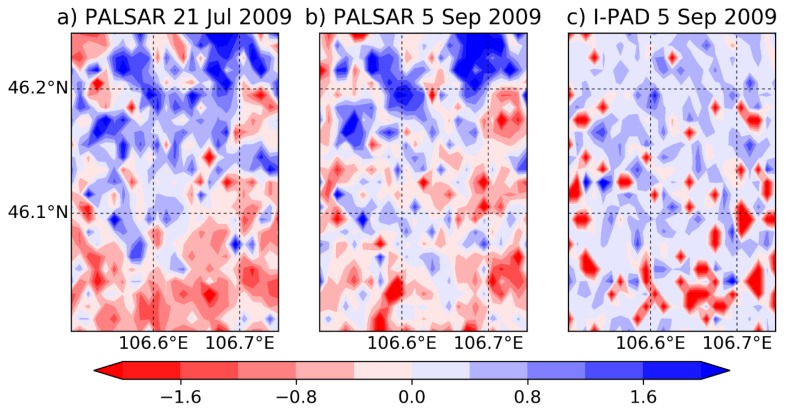
Spatial distributions of normalized surface soil moisture on (**a**) 21 July 2009 obtained by PALSAR, and on 5 September 2009 obtained by (**b**) PALSAR and (**c**) I-PAD.

Figure 5 displays a comparison of the soil moisture distributions on 5 September 2009 obtained by PALSAR and the assimilated model after running for 46 days in Mongolia. The initial soil moisture distribution on 21 July 2009 is also shown in Figure 5a. As can be seen from Figure 5a,b, the northern part of the domain is generally wetter, and the southern part is drier. Figure 5c shows that I-PAD homogenized the distribution of soil moisture due to the use of the uniform rainfall over the domain. Note that since we normalized the soil moisture values, these figures are focused on visualizing relative differences between the grid points which were mainly caused by the optimized hydrological parameters. The differences between the optimized parameters were generated based only on the PALAR soil moisture and MODIS LAI. Thus, it is possible that the optimization result at a grid point was a local optimum considering a large number of the parameters.

**Figure 6:**
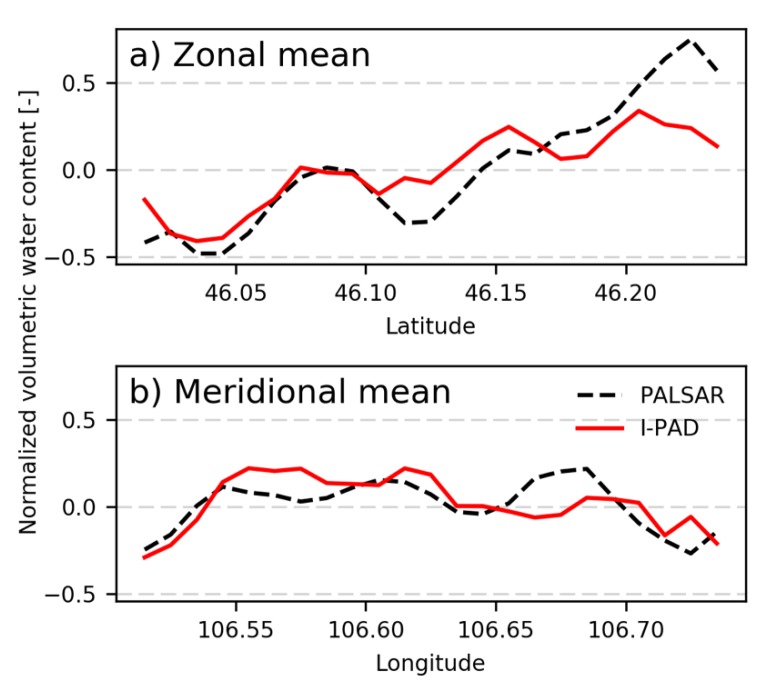
(**a**) Zonal mean and (**b**) meridional mean of normalized surface soil moisture over the study area on 5 September 2009. Black dashed line, PALSAR observation; red line, I-PAD after 40 days run.

Given that the value in a single point may not be reliable, the zonal and meridional averages of normalized soil moisture over the target area at the Mongolia site (blue box in Figure 2a) were analyzed for the results on 5 September 2009 (Figure 6). It is clear from Figure 6a that although I-PAD underestimated soil moisture at the most northern part, it captured the overall trend of the north-south soil moisture pattern. In addition, Figure 6b indicates that I-PAD succeeded in estimating the wetter region in the center and drier regions in the domain edges in the east-west direction. Therefore, it demonstrates that the optimization scheme was able to estimate general patterns of soil texture and hydraulic properties with high-resolution information only from PALSAR soil moisture and MODIS LAI at the Mongolia site.

### 4.2. Little Washita

**Figure 7:**
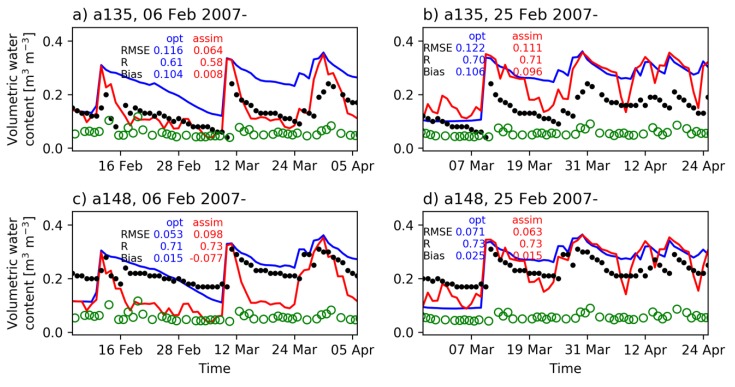
Time series of surface soil moisture (m^3^ m^-3^) from the optimized model (blue), assimilated model (red), observation (black dot), and AMSR-E soil moisture (green circle) at (**a**) a135 from 6 February 2007, (**b**) a135 from 25 February 2007, (**c**) a148 from 6 February 2007, and (**d**) a148 from 25 February 2007 in Little Washita. Each case was simulated 60 days from the initial date. Skill metrics are indicated on each figure as the root-mean-square error (RMSE, m^3^ m^-3^), correlation coefficient (R), and mean bias (m^3^ m^−3^).

Figure 7 shows the time series of soil moisture at two selected observation sites with different starting dates in the Little Washita basin. The AMSR-E soil moisture product (green circles) shows underestimations over this basin which basically results in lowering the level of the model soil moisture estimates. Figure 7a demonstrates that although the R was decreased from 0.61 to 0.58, the assimilation of AMSR-E brightness temperature clearly improved the time series of soil moisture at a135 with the simulation starting on 6 February. The RMSE was almost decreased to a half and the bias was reduced by more than ten times due to the assimilation. However, the simulation starting on 25 February was not as successful as seen in Figure 7b. While it has an overlapping period with the simulation shown in Figure 7a, the correction of soil moisture did not perform the same. This result indicates that I-PAD has a high sensitivity to initial conditions. It reveals that the parameter optimization scheme produces different parameter sets depending on initial PALSAR soil moisture and MODIS LAI. 

**Figure 8:**
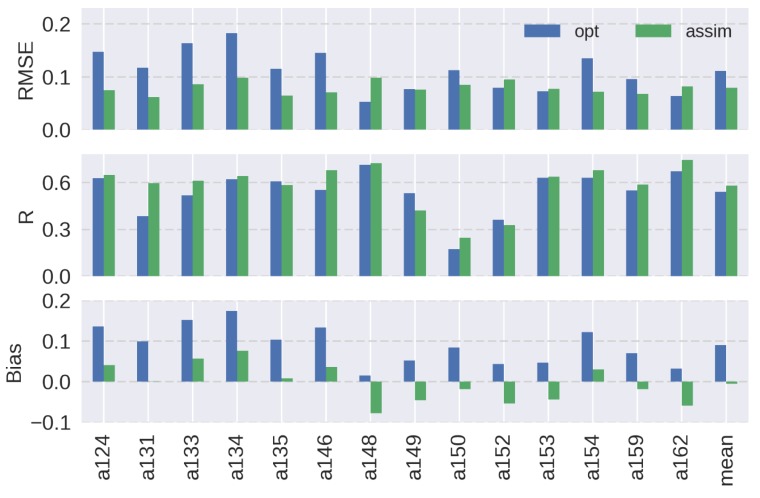
Summary of (top) the root-mean-square error (RMSE, m^3^ m^−3^), (middle) correlation coefficient (R), and (bottom) mean bias (m^3^ m^-3^) for surface soil moisture at each observation site in Little Washita for the simulation starting on 6 February 2007. Blue and green bars show the results of the optimized model and assimilated model, respectively. Average values at all sites are shown on the left.

Figure 7c illustrates that the assimilation may not positively affect soil moisture estimation in some region. The a148 site had the most negative impact among the simulation starting on 6 February (see Figure 8), because it was relatively drier and other sites were relatively wetter than observations during the optimization run. Due to the condition, I-PAD attempted to reduce overall soil moisture according to AMSR-E observations which resulted in the underestimation at a148. However, with respect to the simulation starting on 25 February, the assimilation positively affected soil moisture at a148 in terms of RMSE and bias as shown in Figure 7d. Overall, despite the underestimation of AMSR-E soil moisture, I-PAD positively blends the effects of the parameter optimization using PALSAR soil moisture and the assimilation of AMSR-E brightness temperature to have better estimates than the optimized model and AMSR-E soil moisture estimates.

Figure 8 summarizes the RMSEs, Rs, and biases of soil moisture for the simulation starting on 6 February 2007 at 14 observation stations in Little Washita. The optimized model showed overestimations at all stations, implying the limitations of the parameter optimization scheme. Since 19 parameters were used for the optimization, the reduction of the number of parameters may be required to improve the optimization scheme. Nevertheless, the soil moisture estimation was improved at 10 stations in terms of RMSE, and 11 stations in terms of R and bias by the assimilation. Although the estimation was worsened at some stations, overall the average of the RMSE at all sites was decreased by 29% and the average bias became almost negligible. The most improved site was a131, where the RMSE was almost reduced to a half, the R was increased by 55%, and the bias became close to 0. The sites that showed increases in RMSEs (a148, a152, a153, and a162) had smaller overestimations in soil moisture compared to other sites during the optimization run. As discussed in the results at a148 which had the largest negative impact in RMSE, this condition resulted in having negative impacts by the assimilation based on the linear decomposition of the AMSR-E observations. Among these sites, the Rs at a148, a153, and a162 were improved because the soil moisture variations became larger by the assimilation of AMSR-E observations. At the a152 site, the R was degraded since the variability was relatively small at this site and it was closer to the results of the optimization model. Only the R was degraded at a149 possibly due to the missing data during the peaks in late March to early April at the site. The RMSE and bias were improved at this site.

**Figure 9:**
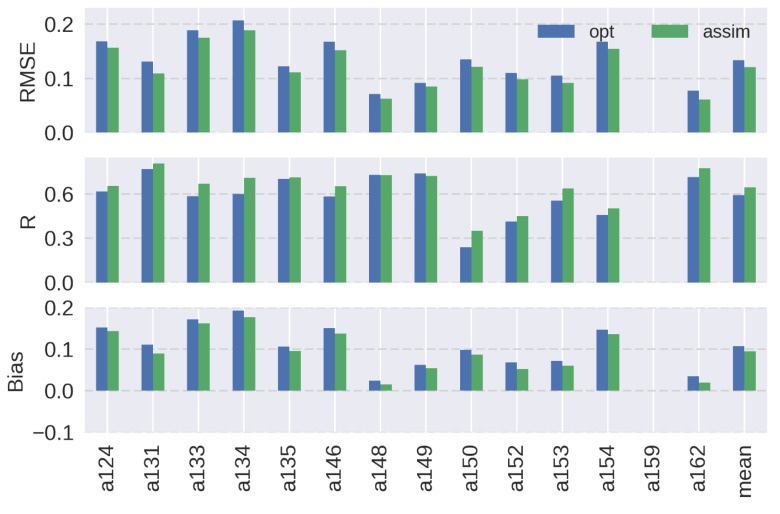
As in Figure 8 but for the simulation starting on 25 February 2007. Note that observation was not available at a159 during this simulation period.

A similar analysis, but with the simulation starting on 25 February 2007, is shown in Figure 9. The soil moisture estimations were improved in terms of RMSE and bias at all sites in this case. Also, the Rs were improved at all sites except a148. However, the simulation yielded higher overall RMSE and bias as compared to the results in Figure 8. These results show the limitation of optimizing model parameters with initial PALSAR soil moisture that has uncertainties generated by the retrieval algorithm. Nevertheless, the assimilation attempted to minimize the overestimations in soil moisture that were caused by the optimized parameters. RMSE and bias were most improved at a162 in this case, where RMSE was decreased by 21% and bias was reduced by 43%. Overall, the results in Figure 8 and Figure 9 confirmed the effectiveness of the simple assimilation algorithm for improving the time series of high-resolution soil moisture.

**Figure 10:**
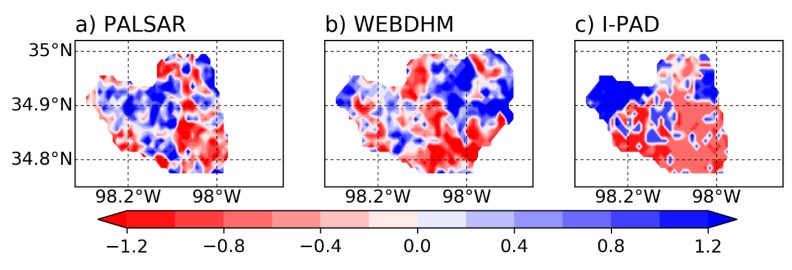
Spatial distributions of normalized surface soil moisture on 2 March 2007 obtained by (**a**) PALSAR (**b**) WEB-DHM, and (**c**) the I-PAD model after a month-long run.

**Figure 11:**
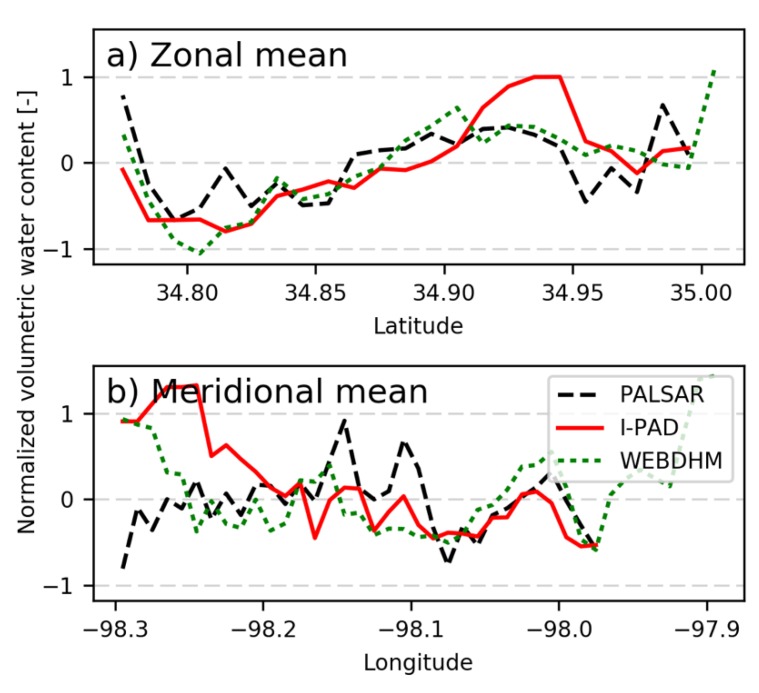
(**a**) Zonal mean and (**b**) meridional mean of normalized surface soil moisture on 2 March 2007. Black dashed line, PALSAR observation; red line, I-PAD; green dotted line, WEB-DHM.

Figure 10 shows the soil moisture spatial distributions of I-PAD, WEB-DHM, and PALSAR on 2 March 2007. I-PAD was simulated for 24 days from 6 February 2007. The standard score normalization was undertaken to compare the distributions estimated by PALSAR, WEB-DHM, and I-PAD. The results of I-PAD and PALSAR are not shown in the whole area because PALSAR observations do not always cover the entire basin. The results in Figure 10c shows that I-PAD tends to produce large anomalies in soil moisture due to the simple assimilation method. To find out the overall trends, we analyzed the zonal and meridional means of normalized soil moisture as displayed in Figure 11. The patterns in Figure 11a indicates that I-PAD simulated zonal mean soil moisture similar to PALSAR and WEB-DHM. However, it overestimated in the western part of the watershed as shown in Figure 11b. Please note that although I-PAD uses PALSAR soil moisture as initial conditions that consider slope effects, the land surface model of I-PAD does not consider lateral flow and ignores the effects of topography, unlike WEB-DHM. Therefore, rainfall was not redistributed correctly in the model regardless of the relatively accurate rainfall inputs from in-situ observations, which could also explain the high spatial variability seen in the results. It should be also noted that WEB-DHM utilized a considerable amount of in-situ forcing (rainfall, solar radiation, relative humidity, air temperature, soil temperature, and wind speed), soil, and land use data, whereas I-PAD only used rainfall as in-situ forcing data. Thus, there are many rooms for improving I-PAD, yet it was able to capture at least the zonal mean trend of soil moisture distributions.

## 5. Discussion

It was demonstrated that I-PAD successfully estimated high-resolution soil moisture at the Mongolia site without the using high resolution land surface parameters and meteorological datasets. The RMSEs of soil moisture time series were below the SMAP mission standard (0.04 m^3^ m^−3^), and the overall trends of soil moisture within the AMSR-E footprint were adequately captured without employing soil texture and hydraulic parameter datasets. The results over the Little Washita basin were, however, not as successful as in the Mongolia site, and some limitations were revealed. Nonetheless, our results are still encouraging compared to the other data assimilation-based studies where the RMSEs ranged from 0.03 to 0.09 m^3^ m^−3^ with the use of high-resolution soil parameter datasets [7]. Uncertainties in this study were caused by initial PALSAR soil moisture, the optimization scheme, the assimilation scheme, and the models.

The hydrological and radiative transfer parameters at each grid point were optimized based on the same AMSR-E observations with different initial soil moisture and LAI. As a result, it was shown that the optimized results were highly dependent on initial conditions. This could be improved by incorporating multiple PALSAR and MODIS observations into the optimization processes. In addition, the number of the optimization parameters can be reduced through selecting parameters that have higher sensitivities to microwave brightness temperature [38]. The selection of parameters would also alleviate the issue of obtaining a local optimum by the optimization.

Although it was shown the I-PAD assimilation scheme improved soil moisture estimation, many limitations were found in the simple assimilation algorithm with the linear decomposition. Future work should use a more sophisticated algorithm to treat the uncertainties in AMSR-E observations correctly and prevent soil moisture to become implausible values. The effectiveness of the frequency bands used in this study also needs to be reconsidered. As we used an L-band SAR, observations by the passive sensors with L-band, such as SMOS and SMAP, need to be assimilated in the model. The simultaneous assimilation of soil moisture and LAI could be also investigated.

Soil moisture is affected by several factors such as soil characteristics, topography, land cover and meteorological forcing across the scale [55]. In this study, the effects of soil texture and vegetation were considered by optimizing model parameters using PALSAR and MODIS high spatial resolution data. However, we only applied I-PAD to the semiarid regions. Future work should investigate the applicability of this algorithm over different vegetation conditions. The radiative transfer model could be improved to be more applicable to vegetated regions such as by incorporating optical sensor observations [56] but it would be challenging to apply the current system over densely vegetated regions because of the small sensitivity of soil moisture to C-band microwave brightness temperature [57].

Obtaining rainfall patterns within the AMSR-E footprint scale is still challenging. Although we used the intense in-situ observed rainfall in Little Washita, such observation networks are rare. To make I-PAD more applicable, it would be desirable to use global products, such as the Global Satellite Mapping of Precipitation (GSMaP) [58] dataset, which has a spatial resolution of 0.1°. We did not use these data in this study as it could be an additional error source without an appropriate bias correction. However, this issue must be resolved for global applications. Furthermore, in-situ soil moisture and roughness measurements were used in the PALSAR soil moisture retrieval. Although we separated the parameters used in the PALSAR retrieval and I-PAD, incorporating the PALSAR retrieval processes in the I-PAD system would be a solution to apply the method without in-situ measurements. In addition, the effects of topography should be included in the land surface model, as indicated by the results in the Little Washita basin. It is necessary to consider lateral flow to accurately simulate the redistribution of rainfall, though the computational efficiency of I-PAD should be improved before such applications.

## 6. Conclusions

Microwave sensors are one of the effective methods to measure surface soil moisture. However, active and passive microwave sensors provide either poor temporal or spatial resolution. This study aimed to produce a synergy between active and passive sensors to estimate high spatial and temporal soil moisture over data-sparse regions. To achieve this goal, we developed a dual-pass assimilation system, named I-PAD, which can incorporate both active and passive data. Soil moisture distributions obtained from PALSAR were used as initial conditions, and brightness temperatures at two frequencies obtained from AMSR-E were assimilated through the RTM in this system. The discrepancy between the spatial resolutions of the model grids and AMSR-E were considered in the assimilation step by assuming linearity in microwave emission. The performance of I-PAD was evaluated at the two lightly vegetated regions at the semiarid CEOP/Mongolia reference site and the Little Washita basin.

It was demonstrated that this active and passive combined approach improved the ability to estimate the soil moisture time variations on a local scale and simulated the general spatial patterns of soil moisture within the AMSR-E footprint with a relatively simple algorithm and without utilizing high-resolution soil parameter datasets. Meanwhile, the limitations of the approach were found and discussed. Future work should investigate on a more robust approach with improved optimization and assimilation schemes using L-band passive observations. 

## Figures and Tables

**Table 1:** Dates of PALSAR observations.

Site	PALSAR Observations
Mongolia	21 July 2009, 5 September 2009, 8 June 2010, 23 July 2010
Little Washita	6 February 2007, 25 February 2007, 2 March 2007

**Figure A1:**
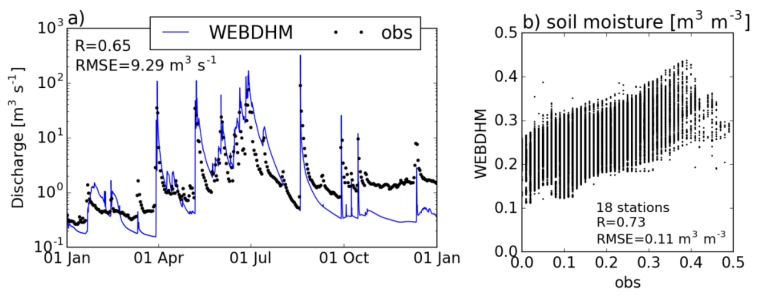
Validations of WEBDHM at the Little Washita basin: (**a**) Temporal variations of discharge by WEBDHM and an in-situ observation site; (**b**) scatter plots of surface soil moisture by WEBDHM and in-situ observation sites. The correlation coefficient (R) and root-mean-square error (RMSE) are indicated in each figure.

## Appendix A

Soil moisture retrieval algorithm from PALSAR observations based on Aida et al. [37], which showed successful results in the Mongolia reference site, is presented in this section. Three PALSAR observation modes, polarimetry (HH/HV/VH/VV), fine-beam single polarization (FBS; HH or VV), and fine-beam dual polarization (FBD; HH/HV or VV/VH), were utilized. To consider the effects of both surface scattering and volume scattering, the integral equation model (IEM) [59] and the dense media radiative transfer model (DMRT) [60] were used. This method first calculated the dielectric properties of a dense medium based on the particle size distribution and its fraction. Then, a second-order Müller matrix was calculated under geometric consideration of a half-space layer using IEM and DMRT. The effects of topography were considered through a slope correction method suggested by Ulander [61]. In this study, direct surface scattering, the coherent component of volume scattering in the specular and non-specular directions, and the incoherent component of volume scattering were included in the Müller matrix. Backscattering could be generally written in this forward model as shown in Equation (A1):

(A1) $$\sigma_{pq}^{o}=f\left(\lambda ,\;p,\;q,\;\alpha ,\;\theta ,\;s,\;l,\;\phi ,\;D\right),$$

where $\sigma_{pq}^{o}$ is the scattering coefficient transmitted at polarization p (vertical or horizontal) and received at polarization q, λ is the frequency, α is the incident angle, θ is the surface volumetric water content (a surrogate for dielectric constant), s is the root-mean-square height, l is the correlation length, ϕ is the soil porosity, and D is the geometric mean diameter of soil particles.

In addition to the sensor dependent parameters, soil moisture (θ), porosity (ϕ), particle size (D), and two surface roughness parameters (s, l) were the factors in backscattering. We assumed that porosity and particle size were invariant within the target areas (scale, about 25 km) during the research period. Furthermore, the time stationarity of the surface roughness parameters was assumed because surface roughness is relatively unchanged under natural conditions, while the spatial heterogeneity of the surface roughness parameters was maintained.

Based on these assumptions, the soil moisture could be estimated from any observation mode if porosity, particle size, and the roughness parameters were known at one time point. To estimate the parameters, we first used a polarimetry image. Using the image, the porosity and particle size were optimized with the observed soil moisture and roughness data at a point location. As porosity and particle size were assumed to be spatially homogeneous, the distributions of the soil roughness parameters and soil moisture were then estimated from the optimized porosity and particle size using the same polarimetry image. Finally, we estimated the soil moisture from FBS and FBD images at different times, assuming that these parameters were invariant with time. A lookup table was prepared beforehand with the forward model to reduce computational cost. All variables were optimized, with the cost function defined as the sum of the square error between the estimated and observed backscattering for each polarization.

## Appendix B

WEB-DHM [54] is a hydrological model that couples Hydro-SiB and GBHM. Following the study by Wang et al. [54], the model was calibrated using discharge data from the United States Geological Survey (USGS) stream gauge, as shown in Figure A1a. Meteorological data (rainfall, solar radiation, relative humidity, air temperature, soil temperature, and wind speed) to run WEB-DHM were obtained from ARS Micronet and Mesonet stations, and interpolated using the IDW method. Soil properties from Gridded Soil Survey Geographic, land use data from USGS, and vegetation data from MODIS were also collected for the model input. Although WEB-DHM had positive biases in the soil moisture estimation in Little Washita, the R with all the ground observation was over 0.7 (Figure A1b). Therefore, in addition to the validation using the PALSAR soil moisture map, the mean of WEB-DHM soil moisture was bias-corrected by linear scaling for validating the spatial distribution.
